# Prevalence of Food-Hypersensitivity and Food-Dependent Anaphylaxis in Colombian Schoolchildren by Parent-Report

**DOI:** 10.3390/medicina57020146

**Published:** 2021-02-05

**Authors:** Carlos Eduardo Beltrán-Cárdenas, Diana María Granda-Restrepo, Alejandro Franco-Aguilar, Veronica Lopez-Teros, Aldo Alejandro Arvizu-Flores, Feliznando Isidro Cárdenas-Torres, Noé Ontiveros, Francisco Cabrera-Chávez, Jesús Gilberto Arámburo-Gálvez

**Affiliations:** 1 Postgraduate Program in Nutrition Sciences, Faculty of Nutrition Sciences, University of Sinaloa, Culiacan, Sinaloa 80019, Mexico; carlos.1.beltran.uacng@uas.edu.mx (C.E.B.-C.); feliznando@uas.edu.mx (F.I.C.-T.); 2 Food Department, Faculty of Pharmaceutical and Food Sciences, University of Antioquia, Medellín, Antioquia 50010, Colombia; diana.granda@udea.edu.co (D.M.G.-R.); afrancoaguilar@gmail.com (A.F.-A.); 3 Postgraduate Program in Health Sciences, Division of Biological and Health Sciences, University of Sonora, Hermosillo, Sonora 83000, Mexico; veronica.lopez@unison.mx (V.L.-T.); aldo.arvizu@unison.mx (A.A.A.-F.); 4 Clinical and Research Laboratory (LACIUS, URS), Department of Chemical, Biological, and Agricultural Sciences (DC-QB), Division of Sciences and Engineering, University of Sonora, Navojoa, Sonora 85880, Mexico; noe.ontiveros@unison.mx

**Keywords:** food allergy, food-dependent anaphylaxis, food allergens, children, epidemiology

## Abstract

*Background and objectives*: The epidemiology of food allergy (FA) and food-dependent anaphylaxis remains unknown in Colombia. Our aim was to estimate by parent-report the prevalence of FA and food-dependent anaphylaxis in a Colombian population of schoolchildren. *Materials and methods:* A printed questionnaire was sent to parents of schoolchildren aged 5–12 years old from Medellín, Colombia in order to collect FA-related data. *Results:* Nine hundred and sixty-nine (969) parents returned the questionnaire with valid responses (response rate, 52.5%). The estimated prevalence rates (95% CI) were: adverse food reactions 12.79% (10.76–15.07), “perceived FA, ever” 10.93% (9.08–13.08), “physician-diagnosed FA, ever” 4.33% (3.14–5.81), “immediate-type FA, ever” 6.81% (5.30–8.58), “immediate-type FA, current” 3.30% (2.26–4.63), and food-dependent anaphylaxis 1.85% (1.10–2.92). The most frequently reported food allergens were milk (1.44%), fruits (0.41%), meat (0.41%), and peanut (0.3%). Sixty-one percent of “food-dependent anaphylaxis” cases sought medical attention, but only eleven percent of the cases reported the prescription of an epinephrine autoinjector. *Conclusions:* FA and food-dependent anaphylaxis are not uncommon among schoolchildren from Colombia. The prescription of epinephrine autoinjectors should be encouraged among health personnel for the optimal management of suspected cases of food-dependent anaphylaxis.

## 1. Introduction

Food allergy (FA) has been reported as the main cause of anaphylaxis during childhood [1,2]. In the last years, the number of children that received medical attention in emergency departments due to FA-related symptoms has increased [3] although the frequency of fatal anaphylaxis has not [4]. The FA prevalence in children has been well documented in developed countries [5,6,7,8,9,10], but just a few Latin American countries have reported data about it [11,12,13,14]. Estimating the prevalence of FA and identifying the suspected foods triggering would be helpful for a better understanding of the impact and burden of the condition in specific populations [15]. To our knowledge, no population-based epidemiological study on FA and food-depend anaphylaxis has been carried out in Colombia in the last twelve years. Therefore, our aim was to conduct a survey-based cross-sectional study to estimate the prevalence of FA in school-aged children from Medellín, Colombia.

## 2. Materials and Methods

### 2.1. Population Survey

A population-based cross-sectional survey was carried out in Medellín, Colombia, during August and September 2019. Parents of school-aged children (aged 5–12 years old) participated in the study. The sampling was carried out by convenience in two private (with high socioeconomic stratum) and four public elementary schools (with low socioeconomic stratum) located in the urban area of Medellín city and geographically distributed across the city. Printed questionnaires and informed consents were distributed among the parents by sending them attached to the children’s homework notebooks. Non-response by the parents was considered when the questionnaire and signed informed consent were not returned after two weeks. The sample size was calculated using an expected prevalence of parent-reported FA of 5.5% with an absolute error of two and a 95% confidence interval. A sample of at least 498 children was considered representative.

### 2.2. Questionnaire

A self-administered Spanish version of a validated questionnaire was culturally adapted and used in this study [11,12,13]. All respondents answered questions related to demographic and clinical information, parental intuition of FA, allergic disease record, and family history of allergic disease. Those reporting positive response to perceived food-related recurrent symptoms answered in-depth questions about the foods that trigger the symptoms, specific food-related recurrent symptoms, time of appearance of the symptoms, number of reactions and treatment, prescription of epinephrine autoinjectors and restricted diets, among others). This study was approved by the “Ethics and research committee” of the nursing faculty of the Universidad de Antioquia (ethic approval number: Acta Nº CEI-FE 2017-16; May 31, 2019).

### 2.3. Definitions

**Figure 1:**
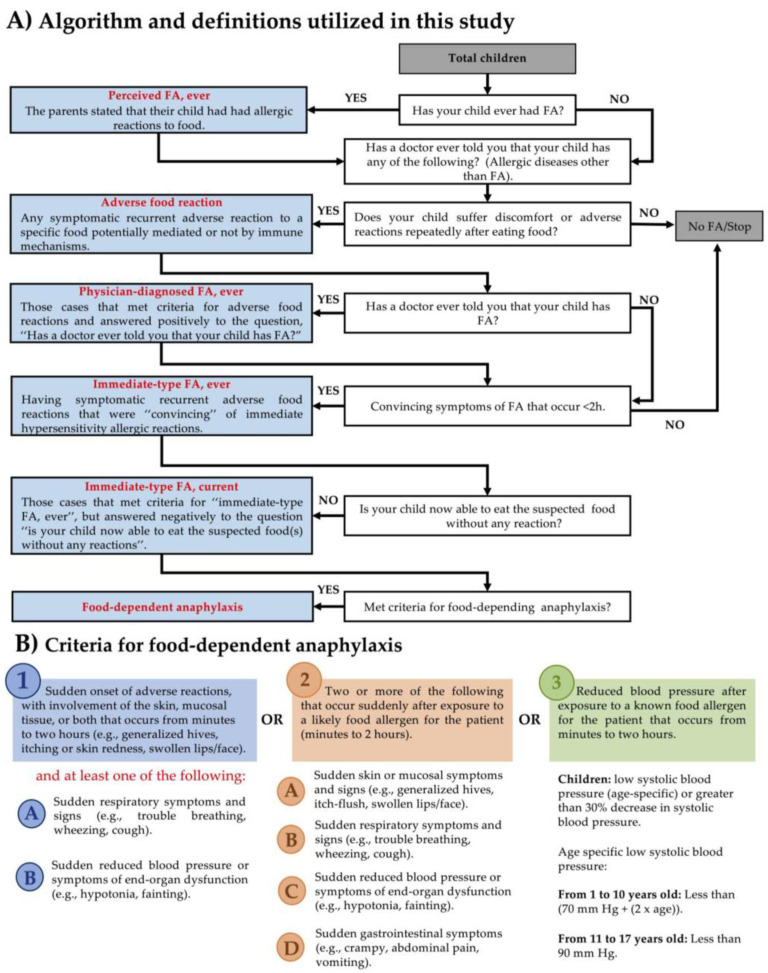
Algorithm, definitions, and criteria for food-dependent anaphylaxis cases utilized in this study. (**A**) Algorithm and definitions utilized in this study. (**B**) Criteria for food-dependent anaphylaxis according to the World Allergy Organization anaphylaxis guidelines [16].

“Perceived FA, ever”, “adverse food reaction”, “physician-diagnosed FA, ever”, “immediate-type FA, ever and current”, and “food-dependent anaphylaxis” were defined as previously described (Figure 1A) [12,13]. Convincing symptoms of immediate hypersensitivity involve the respiratory tract (trouble breathing, itchy throat, throat tightness, wheezing, cough, rhinitis, and nasal congestion), the skin (skin with hives, skin redness, and swelling of lips/face), and the gastrointestinal tract (diarrhea, vomit, abdominal pain), among other symptoms (itchy eyes with redness, low pressure, fainting) that occur within 2 h after food ingestion. Food-dependent anaphylaxis was defined according to the criteria established by the World Allergy Organization [16] (Figure 1B).

### 2.4. Statistical Analyses

Statistical analysis was carried out using GraphPad Prism software, version 8.0 (GraphPad Software, San Diego, CA, USA). For descriptive analyses, categorical variables were described as numbers and percentages. Differences between categorical variables were tested by a two-tailed Fisher’s exact test. Exact odds ratio (OR) and confidence intervals were calculated by Baptista and Pike method. Continuous variables were summarized by mean and range. The OpenEpi software version 3.03a (www.OpenEpi.com, updated 6 April 2013 and accessed 6 December 2020) was used to calculate the prevalence rates. Rates were reported as a rate (95% confidence interval) per 100 inhabitants. A *p*-value <0.05 was considered statistically significant.

## 3. Results

### 3.1. Participants and Demographic Characteristics

The demographic and clinical characteristics of the participants are shown in Table 1. A total of 1846 questionnaires were delivered to schoolchildren’s parents. The response rate was 56.2% (*n* = 1038), but 69 individuals were excluded from the study due to incomplete demographic data (valid response rate, 52.5% (*n* = 969)). The gender ratio was 56.6/43.4 (female/male), and 60.2% of the children were from public elementary schools. Allergic disease history was reported by 43.6% (*n* = 423) of the participants and 21.0% (*n* = 204) reported more than one allergic disease. Allergic rhinitis (20.5%), atopic dermatitis (11.2%), insect sting allergy (11.0%), and asthma (11.0%) were the most commonly reported allergic diseases (Table 1).

### 3.2. Parent-Reported Prevalence Rates Estimations of Adverse Food Reactions and FA

Prevalence estimations are shown in Table 2. The prevalence of “immediate-type FA, current” and “food-dependent anaphylaxis” was 3.30 (95% CI, 2.26–4.63) and 1.85 (95% CI, 1.10–2.92), respectively. A total of 106 (10.93%) parents reported that their children had FA and 42 (4.33%) informed that a physician diagnosed their children. A total of 124 (12.79%) parents reported that their children suffer discomfort or adverse reactions repeatedly after eating a specific food (adverse food reactions, 12.79%, 95% CI, 10.76–15.07), but only 66 reported convincing symptoms of immediate-type FA (immediate-type FA, ever; 6.81%, 95% CI, 5.30–8.58). The prevalence of “food-induced anaphylaxis” was higher in the 9–12 years old children group than in the 4–8 years old group (*p* < 0.05). According to the type of school, both “perceived FA, ever” and “physician-diagnosed FA, ever” prevalence rates were higher in children from private than in children from public elementary schools (7.20% vs. 16.6% and 2.6% vs. 7.0%, respectively; *p* < 0.05 in both cases) (Supplemental material Table S1). Statistical comparisons by gender were not significant (*p* > 0.05) (Supplemental material Table S2). Twenty-one children had convincing symptoms of FA, but the symptoms occurred after 2 h of the ingestion of the suspected food (*n* = 13) or the parents were unaware of the temporality of the symptoms reported (*n* = 8). Of these 21 cases, 15 parents reported restricting the suspected food from the children’s diet.

Children with a parent-report of immediate-type FA, either ever or current, had a significant increased rate of asthma (24.24 vs. 10.07; *p* < 0.01), urticaria (13.63 vs. 4.09; *p* < 0.01), allergic rhinitis (43.93 vs. 18.82; *p* < 0.0001), atopic dermatitis (28.78 vs. 9.96; *p* < 0.0001), insect sting allergy (19.69 vs. 10.40; *p* < 0.05), and pet dander allergy (30.30 vs. 7.53; *p* < 0.0001) than children without FA (Supplementary material Table S3). Furthermore, children with family history of allergic disease were more likely for developing immediate-type FA (mother OR = 3.592 (95% CI, 2.178–5.949); sibling OR = 2.650 (95% CI, 1.590–4.379)). 

### 3.3. Foods Causing Symptomatic Adverse Reactions

**Figure 2:**
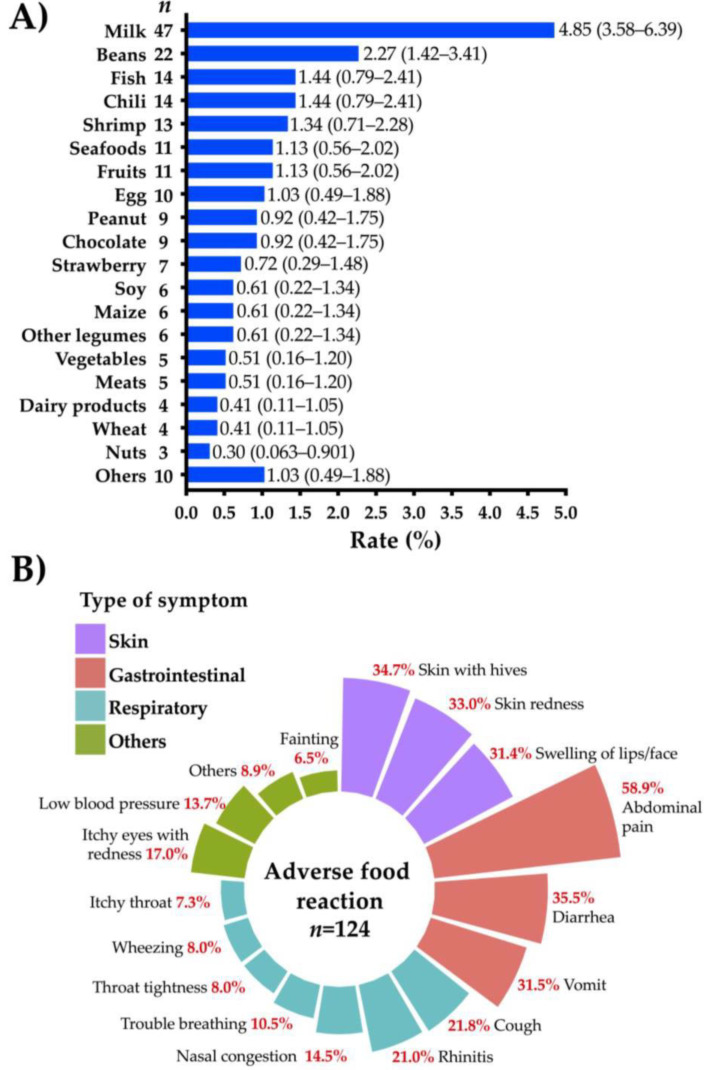
Foods and symptoms associated with adverse food reactions. (**A**) Prevalence of foods associated with adverse food reactions in Colombian schoolchildren (*n* = 969), in brackets are shown 95% confidence intervals; (**B**) prevalence of symptoms in Colombian schoolchildren with reported adverse food reactions (*n* = 124).

More than 20 different foods were reported as the triggers of recurrent adverse reactions (Figure 2A). Milk (4.85%), beans (2.27%), fish (1.44%), and chili (1.44%) were the most commonly implicated foods triggering adverse reactions. The most frequently reported symptoms were abdominal pain (58.9%), diarrhea (35.5%), skin with hives (34.7%), skin redness (31.3%), and vomit (31.5%) (Figure 2B). Fifty-six percent (*n* = 70) of those with adverse food reactions (*n* = 124) sought medical attention.

### 3.4. Common Food Allergens and Clinical Characteristics of FA Cases

**Figure 3:**
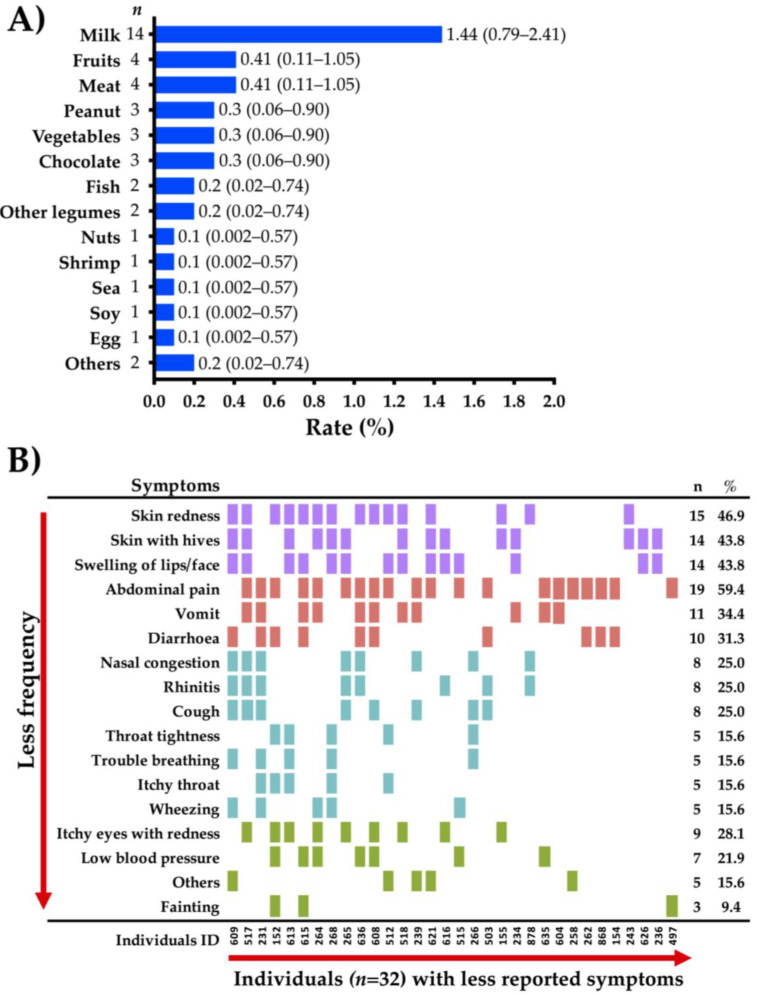
Specific food allergens and symptoms associated with “immediate-type food allergy (FA), current”. (**A**) Prevalence by food of “immediate-type FA, current” in Colombian schoolchildren (*n* = 969), in brackets are shown 95% confidence intervals; (**B**) frequency of specific symptoms in Colombian schoolchildren with “immediate-type FA, current” (*n* = 32).

The reported food allergens and the symptoms associated with food allergic reactions in “immediate-type FA, current” cases are shown in Figure 3. The most frequently reported food allergens were milk (1.44%; 95% CI, 0.79–2.41), followed by fruits (0.41%; CI 95%, 0.11–1.05), meats (0.41%; CI 95%, 0.11–1.05), and peanut (0.3%; CI 95%, 0.06–0.90). Nuts, shrimp, shellfish, soy, and egg were the less frequently reported food allergens (0.1%; CI 95%, 0.002–0.57) (Figure 3A). The symptoms triggered by food allergens in children that fulfilled the criteria for “immediate-type FA, current” mainly affected the gastrointestinal tract (71.8%), followed by the skin (65.6%) and the respiratory tract (53.1%) (Figure 3B). The most frequently reported specific symptoms were abdominal pain (59.4%), skin redness (46.9%), skin with hives (43.8%), swelling of lips/face (43.8%), and vomit (34.4%) (Figure 3B). Five out of six (83.3%) and fifteen out of twenty-seven (55%) children with immediate-type FA to egg or milk, respectively, reported outgrowing their allergy. Fifty-six percent (*n* = 18) of “immediate-type FA, current” cases (*n* = 32) received emergency medical attention. Intravenous or oral hydration (53.8%), antihistamines (34.6%), and inhalers (26.9%) were the most frequently administered treatments. Regarding “food-dependent anaphylaxis” cases (*n* = 18), only 8 (44%) reported a physician diagnosis of FA. The number of allergic reactions ranged from 5 to 10 among “food-dependent anaphylaxis” cases and 11 (61.1%) parents informed that they sought emergency medical attention. Only 2 cases (11.1%) reported the prescription of an epinephrine autoinjector.

## 4. Discussion

The prevalence estimation of “immediate-type FA, current” in Colombian schoolchildren was 3.3% and the main triggers of the condition were milk, fruits, meats, and peanuts. A previous survey that included the Colombian population aged 1–83 years old reported a FA prevalence of 14.9%, fruits/vegetables, seafood, meats, and liquor being the most frequent triggers of the condition [17]. These differences could be explained by the target population and the criteria to define FA cases. Our prevalence estimation of “immediate-type FA, current” is similar to the one reported in Mexican population (3.5% [12]), but it is lower than the prevalence rates estimated in other Latin American countries using the same instrument and evaluating populations with the same characteristics (El Salvador 5.3% and Chile 5.5%) [11,13]. Similar studies conducted in North America (USA 7.6% [5], Canada 7.14% [6]), Europe (6.86% in children aged 0 to 17 years old from all regions of Europe [18], 5.5% in children aged 6 to 12 years old from 10 European countries [8], 1.4 to 3.8% in school-aged children in eight European countries [10]), Asia (Taiwan 7.7% [19], Korea 4.06% [20], and Vietnam 8.9% [21]), and the Middle East (Lebanon 4.1% [22]; United Arab Emirates 8.0% [23]) have reported higher prevalence rates than the one reported in the present study. Various factors can influence the prevalence estimations such as the instruments used, the age range of the target population, and the criteria for defining allergy cases [15]. Furthermore, differences in FA prevalence among different regions can be attributed to cultural aspects, eating patterns, genetic inheritance, and socioeconomic factors [24]. Despite differences among the FA prevalence rates estimated in the present and other studies, the results highlight that food hypersensitivities are common in Colombian schoolchildren.

Children from private elementary schools had a higher prevalence rate of “perceived FA, ever” and “physician-diagnosed FA, ever” than those from public ones. This could be attributed to socioeconomic aspects such as economic income and access to health services. Independently of the type of school (public or private), a significant association of FA with asthma, atopic dermatitis, urticaria, allergic rhinitis, animals, and insect sting allergy was observed. These results support the notion that prevalence rates of self-reported FA and physician-diagnosed FA are more commonly reported in high-income than in low-income populations [21] and that there is a significant association of FA with other atopic diseases [25].

The foods triggering FA reactions can vary from country to country and in some cases even within the same country’s regions [15,24]. The main triggers of FA reactions in Colombian schoolchildren were milk, fruits, meat, and peanut. Milk is a well-known food allergen and one of the main allergens reported in schoolchildren around the world (Canada 2.23% [6]; USA 1.9% [5]; Europe 1.9% [8]; El Salvador 1.7% [13]). Fruits have only been reported as the main allergens in countries of the Mediterranean area, the Middle East, and Asia [22,23,26], and now in Colombian schoolchildren. Studies focused on cultural aspects, eating patterns, genetic inheritance, and socioeconomic factors are needed for an in-depth explanation of the high prevalence of allergy to fruits in Colombians and people from Mediterranean area, Middle East, and Asia. Regarding allergy to mammalian meat, this is an IgE-mediated hypersensitivity triggered after the recognition of the carbohydrate epitope of mammalian meat alpha-Gal (galactose-alpha-1,3-galactose) [27] and other epitopes in allergenic proteins from cow, pig, and horse [28,29]. The sensitization may occur by inhalation, orally, or through the skin [28,29]. The alpha-Gal sensitization can occur by skin route through tick bites [30,31]. Notably, the presence of 26 species of ticks of the genus *Amblyomma* has been reported in Colombia and at least 7 of these species are common in the Medellín area (on Antioquia department) [32]. However, a potential high prevalence of alpha-Gal sensitization or meat allergy due to the alpha-Gal allergenic epitope will require evaluations with objective diagnostic criteria. At the moment, our results highlight the need for future studies to evaluate alpha-gal sensitization in this population. 

Peanut is commonly reported as the main trigger of FA in Western countries (2.2% in USA [5]; 1.77% in Canada [6]; 1.9% in Australia [33]). Furthermore, in some Latin American countries (Chile 1.1% [11]; El Salvador 0.98% [13]) it is the main cause of anaphylaxis in children [34]. The present study shows that the prevalence of peanut allergy in Colombian schoolchildren is uncommon (0.3%) compared to the prevalence rates reported in the USA (2.2%), Canada (1.77%), and Australia (1.9%) [5,6,33]. This difference in prevalence rates could be influenced by the low consumption of peanuts in Colombia (0.8 g/day/person) [35] compared to the consumption in those developed countries (7.3 g/day/person) [36]. Regarding egg, this food is highly consumed in Colombia (291 units/year/person) [37] and it is one of the most commonly reported allergens in some industrialized countries [5,33,38]. However, egg was one of the less frequently reported allergens (0.1%) among Colombian schoolchildren. The low prevalence of egg allergy in Colombian schoolchildren can be explained by the highly reported egg tolerance development rate (83%; 5 out of 6 children), which has been reported by others [39]. The heterogeneity observed in the FA triggering foods and FA prevalence rates across different countries highlights the need to direct efforts to better understand the impact and burden of FA in different populations. 

Anaphylaxis is a serious, rapid-onset, potentially fatal systemic allergic reaction, and recent reports show that foods are the most frequent trigger of it in children [40]. Notably, an increase of up to 9.7% in fatal food anaphylaxis rates has been reported in some countries [41]. In the present study, more than half of the cases of “immediate-type FA, current” reported a reaction compatible with food-dependent anaphylaxis (18 of 32 cases). Although epinephrine/adrenalin is indicated as the first-line treatment for anaphylaxis [16], only one of the affected children was treated with intramuscular adrenaline and only two cases reported the prescription of an epinephrine autoinjector. Other Latin American studies have reported the low prescription of epinephrine devices [11,12,13]. This last could be influenced by the previous lack of guidelines for the management of anaphylaxis and the lack of epinephrine auto-injectors for sale in most Latin American countries [2]. Recently (March 2019), guidelines for the management of anaphylaxis for the Latin America area were published and were endorsed by the “Colombian Association of Allergy, Asthma and Immunology” [42]; however, there is still a limited availability of commercial epinephrine auto-injectors in Colombia [2]. These circumstances suggest that it is highly probable that food-dependent anaphylaxis is not optimally managed in Colombia and highlights the need to improve the knowledge of health personnel to optimize the treatment and long-term management of this allergic condition.

The main strengths of our study are its population-based design and the inclusion of data from children geographically distributed across Medellín city, which allowed us to collect data from neighborhoods with different socioeconomic levels. Furthermore, it should be noted that this is the only study that has reported prevalence estimations in Colombian population in the last decade. Certainly, we should acknowledge that our study has some limitations. Firstly, the sampling was carried out in only one city in Colombia. Incorporating data from other regions of the country would have allowed us to establish regional profiles of the FA-triggering foods. Secondly, the immediate-type FA cases were not confirmed with clinical test (e.g., specific IgE, skin prick test, or oral challenges), and epidemiological studies by self-report may overestimate the prevalence of FA, e.g., food intolerances are limited to digestive problems. Despite these limitations, our study generates important data about the prevalence, clinical manifestations, and management of FA in the Colombian population and serve as a reference point for further studies based on objective diagnostic criteria.

## 5. Conclusions

To our knowledge, the present study is the first in applying strict criteria to define and report FA and food-dependent anaphylaxis prevalence rates in Colombia in the last twelve years. Our data suggest that FA and food-dependent anaphylaxis are not uncommon among schoolchildren from Colombia and that food-dependent anaphylaxis is not optimally managed by health personnel probably due to the unavailability of epinephrine autoinjectors.

## Figures and Tables

**Table 1:** Demographic and clinical characteristics of the study population.

Variable		
Mean Age in Years (Range)		8.75 (5–12)
		*n* (%)
Gender	Female	547 (56.4)
	Male	422 (43.6)
Elementary school	Public	583 (60.2)
	Private	386 (39.8)
Known allergic disease		
Allergic rhinitis		199 (20.5)
Atopic dermatitis		109 (11.2)
Insect sting allergy		107 (11.0)
Asthma		107 (11.0)
Allergic conjunctivitis		97 (10.0)
Pet dander allergy		88 (9.1)
Drug allergy		53 (5.5)
Urticaria		46 (4.7)
Anaphylaxis		10 (1.0)

**Table 2:** Prevalence estimations.

Assessment	Number of Cases	Prevalence % (95% CI)			*p*
		4–8 Years, *n* = 430	9–12 Years, *n* = 539	Total, *n* = 969	
Adverse food reactions	124	11.62 (8.75–15.04)	13.72 (10.94–16.93)	12.79 (10.76–15.07)	0.383
Perceived FA, ever	106	11.35 (8.55–14.78)	10.57 (8.10–13.48)	10.93 (9.08–13.08)	0.68
Physician-diagnosed FA, ever	42	5.11 (3.23–7.64)	3.71 (2.28–5.67)	4.33 (3.14–5.81)	0.341
Immediate-type FA, ever	66	5.81 (3.79–8.46)	7.60 (5.51–10.18)	6.81 (5.30–8.58)	0.3055
Immediate-type FA, current	32	2.79 (1.45–4.82)	3.71 (2.28–5.67)	3.30 (2.26–4.63)	0.4732
Food-dependent anaphylaxis	18	0.69 (0.14–2.02)	2.78 (1.56–4.54)	1.85 (1.10–2.92)	0.016

## Data Availability

The data presented in this study are available within the article and the supplementary material.

## Supplementary Materials

The following are available online at https://www.mdpi.com/1010-660X/57/2/146/s1, Table S1. Prevalence estimates stratified by type of school; Table S2. Prevalence estimates stratified by sex; Table S3. History of other allergic diseases between “FA, ever” and non-FA cases.
