# Supplementary material for: Prevalence of Food-Hypersensitivity and Food-Dependent Anaphylaxis in Colombian Schoolchildren by Parent-Report
Source: Medicina (Kaunas). 2021 Feb 5;57(2):146. doi: 10.3390/medicina57020146 (PMC7915673; doi:10.3390/medicina57020146)
Supplement: Supplementary file 1 [file medicina-57-00146-s001.pdf]

Supplemental Material

Table S1. Prevalence estimates stratified by type of school.

Assessment	Number of reported cases	Prevalence % (95% CI)		*p*
		Public=583	Private=386	
Adverse food reactions	124	11.83 (9.32-14.74)	14.24 (10.92-18.14)	0.281
Perceived FA, ever	106	7.20 (5.24-9.61)	16.58 (13.01-20.67)	**<0.0001**
Physician-diagnosed FA, ever	42	2.57 (1.44-4.20)	6.99 (4.66-10.01)	**0.0012**
Immediate-type FA, ever	66	6.51 (4.65-8.83)	7.25 (4.87-10.31)	0.6967
Immediate-type FA, current	32	3.25 (1.97-5.04)	3.36 (1.80-5.69)	>0.9999
Food-induced anaphylaxis	18	2.22 (1.19-3.78)	1.29 (0.42-2.99)	0.3399

FA: Food allergy

Table S2. Prevalence estimates stratified by sex.

Assessment	Number of reported cases	Prevalence % (95% CI)		*p*
		Male=422	Female=547	
Adverse food reactions	124	11.84 (8.92-15.32)	13.52 (10.77-16.68)	0.4974
Perceived FA, ever	106	9.71 (7.06-12.95)	11.88 (9.29-14.89)	0.3007
Physician-diagnosed FA, ever	42	3.55 (2.0-5.79)	4.93 (3.27-7.10)	0.3414
Immediate-type FA, ever	66	6.87 (4.65-9.72)	6.76 (4.80-9.20)	>0.9999
Immediate-type FA, current	32	2.84 (1.47-4.91)	3.65 (2.24-5.59)	0.5875
Food-induced anaphylaxis	18	2.13 (0.97-4.0)	1.64 (0.75-3.10)	0.6355

FA: Food allergy

Table S3. History of other allergic diseases between "FA, ever " and Non-FA cases"

Allergic disease	FA,ever (n=66) n (%)	Non-FA (n=903) n (%)	p	***Odds ratio (95% CI)***
Asthma	16 (24.24)	91 (10.07)	**0.0016**	2.855 (1.566 - 5.189)
Urticaria	9 (13.63)	37 (4.09)	**0.0026**	3.696 (1.678 - 7.733)
Allergic rhinitis	29 (43.93)	170 (18.82)	**<0.0001**	3.379 (2.044 - 5.629)
Anaphylaxis	1 (1.51)	9 (0.99)	0.5078	1.528 (0.1372 - 9.494)
Atopic dermatitis	19 (28.78)	90 (9.96)	**<0.0001**	3.652 (2.045 - 6.393)

Insect sting allergy	13 (19.69)	94 (10.40)	**0.0386**	2.111 (1.104 - 3.891)
Pet dander allergy	20 (30.30)	68 (7.53)	**<0.0001**	5.339 (2.994 - 9.504)
Allergic conjunctivitis	9 (13.63)	88 (9.74)	0.2904	1.462 (0.7074 - 3.067)
Drug allergy	7 (10.60)	46 (5.09)	0.083	2.210 (0.9176 - 4.934)
